# Local illness concepts and their relevance for the prevention and control of malaria during pregnancy in Ghana, Kenya and Malawi: findings from a comparative qualitative study

**DOI:** 10.1186/1475-2875-12-257

**Published:** 2013-07-22

**Authors:** Arantza Menaca, Christopher Pell, Lucinda Manda-Taylor, Samuel Chatio, Nana A Afrah, Florence Were, Abraham Hodgson, Peter Ouma, Linda Kalilani, Harry Tagbor, Robert Pool

**Affiliations:** 1 Centre de Recerca en Salut Internacional de Barcelona (CRESIB), Hospital Clínic-Universitat de Barcelona, Barcelona, Spain; 2 Departamento de Antropología Social, Universidad Complutense de Madrid, Madrid, Spain; 3 Centre for Social Science and Global Health, University of Amsterdam, Amsterdam, The Netherlands; 4 College of Medicine, University of Malawi, Blantyre, Malawi; 5 Navrongo Health Research Centre, Navrongo, Ghana; 6 Department of Community Health, School of Medical Sciences, Kwame Nkrumah University of Science and Technology, Kumasi, Ghana; 7 The Kenya Medical Research Institute (KEMRI) and Centers for Disease Control and Prevention (CDC) Research and Public Health Collaboration, Kisumu, Kenya

**Keywords:** Malaria, Pregnancy, Qualitative methods, Local disease concepts, Africa

## Abstract

**Background:**

In sub-Saharan Africa, the burden of morbidity and mortality linked to malaria during pregnancy (MiP) is significant and compounded by its unclear symptoms and links with other health problems during pregnancy. Mindful of the biomedical and social complexity of MiP, this article explores and compares local understandings of MiP and their links with other pregnancy-related health problems.

**Methods:**

A comparative qualitative study was undertaken at four sites in three countries: Ghana, Malawi and Kenya. Individual and group interviews were conducted with pregnant women, their relatives, opinion leaders, other community members and health providers. MiP-related behaviours were also observed at health facilities and in local communities.

**Results:**

Across the four sites, local malaria concepts overlapped with biomedically defined malaria. In terms of symptoms, at-risk groups, outcomes and aetiology of malaria during pregnancy, this overlap was however both site-specific and partial. Moreover, the local malaria concepts were not monolithic and their descriptions varied amongst respondents. The symptoms of pregnancy and malaria also overlapped but, for respondents, symptom severity was the distinguishing factor. Malaria was generally, though not universally, perceived as serious for pregnant women. Miscarriage was the most widely known outcome, and links with anaemia, low birth weight and congenital malaria were mentioned. Nonetheless, amongst many potential causes of miscarriage, malaria was not recognized as the most important, but rather interacted with other pregnancy-related problems.

**Conclusions:**

Given the overlap of common pregnancy problems with the symptoms of malaria, and the limited association of malaria with its main outcomes, a comprehensive antenatal care programme is the most appropriate strategy for the provision of health education, prevention and treatment for MiP. Variations in locally shared understandings of MiP must however be taken into account when designing and promoting MiP intervention strategies.

## Background

In endemic regions of sub-Saharan Africa, malaria in pregnancy (MiP) is a major preventable cause of maternal morbidity and poor birth outcomes [1]. Current recommended strategies for MiP in sub-Saharan Africa include appropriate case management, intermittent preventive treatment (IPTp) with sulphadoxine-pyrimethamine (SP), and insecticide-treated bed nets (ITNs) [2]. These strategies, however, must be delivered in a context-specific way that takes into account changing levels of transmission and drug resistance [3]. Nonetheless, despite the efforts and the progress made over the last decade, the coverage of IPTp and ITNs amongst pregnant African women is still inadequate [4]. In response to the recognized burden of MiP-related morbidity and mortality and to address the ongoing challenges of prevention and control, the MiP Consortium, comprised of 47 partner institutions in 32 countries, is currently conducting a wide range of research activities in Africa, Asia and Latin America [5]. This article draws on the results of an anthropological programme of research that forms part of the consortium’s Public Health Impact group.

The overall goal of the MiP Consortium’s anthropological programme is to contribute to the development of appropriate MiP interventions by gaining an in-depth understanding of MiP-related attitudes and behaviours at an individual and social level. A review of previous research [6] identified four broad topics that influence the uptake of MiP interventions: concepts of malaria and risk in pregnancy; attitudes towards malaria prevention and treatment; perceptions of antenatal care (ANC) services; and structural factors. These four themes have been widely explored by the MiP Consortium’s anthropology programme and this article focuses on the local concepts of malaria and risk in pregnancy. Additional articles will focus on attitudes towards malaria prevention and treatment interventions, and perceptions of ANC [7]. The analysis of the structural factors affecting delivery and uptake of MiP interventions is however integral to all these articles.

To ensure a thorough analysis of their impact on health-seeking practices, local illness concepts must be studied together with their relationship with biomedical models of disease. Biomedical understandings of MiP are particularly complex and this is apparent across three domains, which are all relevant for health-seeking behaviour: symptoms, risk groups and disease outcomes.

### Symptoms

Although in areas of intense stable transmission MiP has been commonly considered asymptomatic, studies have shown that it is often symptomatic [8] and its symptoms are non-specific – history of fever, headache, malaise, arthromyalgias, vomiting and fatigue. Therefore a blood test (optical microscopy or rapid diagnostic test (RDT)) is necessary to confirm diagnosis [9].

### Risk groups

Not all pregnant women in Africa are equally vulnerable to MiP. In areas of high, stable malaria transmission, MiP is more prevalent and associated with worse outcomes among women experiencing their first or second pregnancy (frequently adolescents) [1,10]. However, in low, unstable transmission areas, MiP is usually symptomatic, acute and affects all parities equally [8].

### Outcomes

Multiple medical studies have demonstrated that MiP is associated with severe maternal anaemia, pregnancy loses, low birth weight (LBW), congenital malaria and perinatal and infant mortality [1,11]. More than one quarter of cases of severe anaemia and one fifth of LBW cases are attributable to malaria in areas of stable high malaria transmission [1]. Except in the case of congenital malaria, which is rarely associated with clinical disease [12,13], all the other outcomes are not univocally associated with malaria: there are a range of different aetiological factors for anaemia in pregnancy [14-16], stillbirths [17], LBW [18] and perinatal and infant mortality [19,20].

The biomedical complexity of MiP - and particularly the often multifactorial relationship between the disease, its symptoms and outcomes - therefore poses challenges for health education and appropriate disease management. Furthermore, in many settings, malaria is also a socially and culturally complex disease. Indeed, previous qualitative research has demonstrated that, all over Africa, local understandings of malaria are varied and often diverge from the biomedical model [21]. A number of studies have also illustrated how these understandings are widely influenced by contact with biomedical health services and health education [22,23]. The anthropological research has shown that local understandings of diseases are generally not systematic, closed nor static: they depend on experiences and change over the time. Moreover, they are not monolithic or consistent among different members of a population [22,24,25]. Anthropological literature also demonstrates that local health concepts cannot be viewed in isolation, but rather that they are related to one another in semantic and experiential networks [22,26]. It is, therefore, necessary to direct attention to how MiP is associated with its symptoms and effects, and to study MiP in the context of general understandings of pregnancy [6].

Mindful of both the biomedical and *social* complexity of MiP, this article analyses local perceptions of malaria and risk during pregnancy with the objective of identifying: 1) the most (culturally) appropriate framework for the design and implementation of MiP interventions, and 2) contradictions between biomedical and local understandings of MiP that could be addressed in health education.

## Methods

The findings presented in this article are drawn from a comparative qualitative study at four sites in three different countries. A multidisciplinary team (made up of social scientists and biomedically trained researchers) based in Ghana, Malawi, Kenya and Spain carried out the study.

### Settings

The study incorporated one country from each of the three main regions of sub-Saharan Africa: Ghana in West Africa, Kenya in East Africa and Malawi in Southern Africa. Two sites with important regional specificities were selected in Ghana for several reasons: to collect data in at least one site of each of the MiP Consortium’s main treatment and prevention activities; to include areas with different patterns of malaria transmission; and to examine intra- as well as inter-country variation.

In central Ghana, fieldwork was conducted in two districts of the Ashanti Region: Ejisu Juaben and Ahafo Ano South. In both districts, agriculture is the main productive activity, and there is a significant proportion of internal migrants, in addition to the majority ethnic group, the Asante [27]. At this site, malaria transmission is moderately high and occurs throughout the year with peaks during the rains in May to October [28]. In each district, data collection was conducted at the district hospitals, two to three health centres, and several smaller clinics.

In northern Ghana, Upper East Region, the fieldwork sites were located in Kassena-Nankana District. This area is part of the Sahel and experiences only one annual rainy season during which people grow millet, maize and vegetables for subsistence. During the rest of the year, part of the population migrates to other regions. The Kassena and the Nankani make up almost 90% of the local population [27]. Here, malaria transmission is hyperendemic: transmission takes place year-round but there is a seasonal pattern with a peak that coincides with the major rains (May to October) and low rates of infection during the dry season [29]. Data were collected at a district hospital in Navrongo, the district capital, and outreach community-based services, which are common throughout the area.

Fieldwork also took place in Chikwawa and Blantyre Districts, southern Malawi. The main ethnic groups in Blantyre District are Chewa and Yao, whereas, in Chikwawa District, they are Manganja and Sena. Most of the women in the area cultivate crops for subsistence and sale at the market. Both districts are in areas of intense, year-round malaria transmission [30]. Fieldwork took place at three hospitals, and six healthcare centres that provide ANC services to the women of these areas.

Finally, in Kenya, fieldwork was carried out in Siaya District, Nyanza Province, where the principal ethnic group, the Luo, make up over 95% of the population. Livelihood activities include subsistence farming of maize, sorghum, millet and cassava. Due to the relatively limited employment opportunities, migration to urban centres is common, particularly to Kisumu, the nearest city. Malaria is endemic and transmission year-round [31] with the greatest disease burden borne by children and pregnant women. Data were collected at the district hospital and smaller health facilities where ANC is delivered.

### Data collection

An anthropological approach was taken to data collection and this entailed a long phase of fieldwork, a wide range of data collection activities, including free-listing and sorting, in-depth interviews, focus group discussion and participant observation, the use of narrative and observational tools, and a flexible, reflexive and iterative process of tool design, data collection and analysis.

Fieldwork was carried out between April 2009 and August 2011, and lasted from one year in Malawi to more than two years in central Ghana. Fieldworkers spent extended periods of time in the communities where data were collected and recorded their experiences of participant observation in field diaries. Participant observation entailed various activities, which depended on the context: for example, in health care facilities, this often involved sitting with the women waiting for consultations, observing interactions with health staff and informally chatting, whereas, in the communities, this could entail assisting with basic chores. In the first phase, at each site, using free-listing and sorting exercises, the research team explored the main problems that pregnant women experience. Later interviews and group discussions were conducted, several women (case studies) were followed and interviewed several times over the course of their pregnancies (see Table 1 for further details), and observations were carried out in the communities and at local health facilities. The research team interacted with informants in their chosen language (English and various local languages). At all sites, in-depth interviews and group discussions were recorded, transcribed verbatim and then (if necessary) translated into English by fieldworkers.

**Table 1:** Respondents

**Method**	**Respondent type**	**Central Ghana**	**Northern Ghana**	**Kenya**	**Malawi**	**Total**
Free listing and sorting	Community members	12	16	17	24	**59**
	Pregnant women	10	10	7	11	**38**
	Health providers*	10	11	6	5	**32**
In-depth interviews	Pregnant women	84	64	69	68	**285**
	Health providers*	33	34	17	21	**105**
	Relatives	26	29	20	16	**91**
	Opinion leaders	12	12	10	12	**46**
Case studies	Pregnant women	19	18	12	18	**67**
Focus group discussions	Community members	10	16	9	16	**51**

*Includes health care staff involved with the provision of ANC at health facilities and TBA working in the communities.

Communication between staff based at the research sites and in Barcelona was frequent and complemented by quarterly field visits. During these visits, members of the research team reflected on the data collection process, discussing at length preconceptions, unexpected findings, contradictions, doubts and fieldwork dilemmas. The quarterly field visits also allowed the Barcelona based social science team to participate in data collection and provide on-going training.

Using a range of data collection tools in combination with the flexible, iterative and reflexive research approach meant that perceptions of the relationship between pregnancy and malaria could be explored from several perspectives. Interviews started with broad research questions related to pregnancy, later, they focused on the problems and diseases that concern pregnant women, and ended with questions about MiP. In contrast, focus group discussions started with general questions about malaria, focusing later on groups particularly vulnerable to malaria and finalizing with MiP. Miscarriage, stillbirths, pre-term deliveries, birth weight and anaemia – and their causes – were also themes explored during interviews. Data collection and analysis were carried out in parallel allowing the incorporation of emerging themes in the design of the tool, and questions’ .

Several measures were taken to reduce the impact of potential sources of bias and ensure the reliability of findings. To limit the possible influence of individual researchers on the study results, data collection was carried out by at least two team members at each site (a fieldworker and social scientist in central Ghana and Kenya, and, due to higher staff turnover, four fieldworkers and a social scientist in Malawi, and five fieldworkers and a social scientist in northern Ghana). Moreover, employing a range of data collection techniques ensured that findings could be triangulated. By carrying out interviews with a variety of respondents (Table 1) and observations in a range of local contexts (healthcare facilities and community spaces), a range of perspectives were explored and incorporated into the study findings.

### Respondents

Five main categories of participants were interviewed (Table 1): pregnant women, their relatives, community members (men and women), opinion leaders and healthcare providers. Purposive sampling was used to ensure that respondents with a wide range of experiences participated in the study. Married and unmarried pregnant women of a range of ages (under 18 to over 60 years), parities (zero to over seven) and gestational ages (zero to nine months) from across the different communities (within the field sites) were interviewed. Relatives included mainly mothers, mothers-in-law and husbands of the pregnant women. The sample of opinion leaders was made up of religious leaders, traditional and political authorities, and women in the local communities. Finally, ANC staff, pharmacists and drug sellers, traditional birth attendants and other relevant healers were interviewed at each site. Respondents were identified in ANC clinics and via contacts in the local communities that strengthened as fieldwork progressed. The final number of participants was a result of the directed sampling and the point of saturation, whereby no further novel insights were identified from interviews.

### Data analysis

At each site, a first phase of data analysis ran in parallel to data collection. Using Atlas.ti 6, flexible codebooks were developed and revised using a combination of established categories based on the original research questions and themes that emerged from the data. The preliminary results obtained from this site-specific analysis were compared and discussed amongst the members of the team in periodic meetings throughout data collection. In a second phase, data associated to the codes relevant to malaria in pregnancy perceptions, were extracted, collated and discussed between authors one and two, looking at the similarities, differences and variations between and within the different sites.

### Ethics statement

Overall ethics clearance was obtained from the Clinical Research Ethics Committee, Hospital Clinic-University of Barcelona. Separate local ethics clearance was obtained at each site: in Ghana, clearance was obtained from the Institutional Review Board of the Navrongo Health Research Centre, Navrongo and the Committee on Human Research Ethics, Kwame Nkrumah University of Science & Technology, Kumasi; in Kenya, clearance was obtained from the Institution Review Board of Centers for Disease Control and Prevention, Atlanta and from the National Ethics Review Committee, Kenya Medical Research Institute, Nairobi; and in Malawi, clearance was obtained from the College of Medicine Research and Ethics Committee. As approved by all ethics review committees and institutional review boards, informed consent was obtained orally from study participants. Oral rather than written informed consent was obtained because the study procedures posed minimal risk to study participants and to avoid the possible negative influence of a written consent on rapport between researchers and respondents. With the agreement of participants, oral consent was recorded prior to each interview or focus group.

## Results

### Terms, symptoms and causes

In the context of pregnancy, the meanings of local terms that were translated as “malaria” or used to describe malaria-related illnesses coincided only partially with biomedically defined malaria. Hence in some instances, respondents described having *malaria*, which health professionals did not consider to be malaria. At other times however health professionals diagnosed malaria, which the pregnant woman did not recognize as such because it did not coincide with her understanding of the overlapping local illness concept. In this article, italicized *malaria* refers to these locally defined illness concepts across the four sites – encapsulating the specific causation, symptoms and outcomes associated with each illness – and malaria (not italicized) to refer to the biomedically defined disease. The terms used to describe these various *malarias*, as opposed to the concept as a whole, are placed in speech marks (except in quotations).

The terms used to label illnesses that overlapped with malaria originated from local languages and English and, at each site, more than one term was often used. In Malawi, and in northern Ghana respondents mainly used the local terms “malungo” (Chichewa), “paa” (Kassem) and “poa” (Nankam). At both sites, although less frequently, the terms “malaria” and “fever” were also used. In Kenya and central Ghana, the situation was the reverse: respondents generally made use of the English words “malaria” (in Kenya and central Ghana) and “fever” (in central Ghana). In central Ghana, a minority of respondents used local language terms: “whuraye” (whiteness), or occasionally “ebunu” (greenish vomit) or “tiridi” (yellowish eyes).

These terms were used to describe illnesses with a range of symptoms, such as fever, vomiting (generally producing liquid of a yellowish colour), weakness/fatigue, general body pain, headache, coldness/shaking, joint pains, abdominal/stomach pains, diarrhoea, loss of appetite, dizziness, yellow eyes/urine/faeces, a bitter taste, thirst, paleness, breathlessness, swollen legs, and rashes/itching. The majority of these symptoms were common across the four sites and coincide with the (albeit broad) clinical description of malaria. Others, such as rashes or itching, are not recognized as clinical symptoms of malaria but were reported in northern and central Ghana, and also in Kenya where one respondent diagnosed her own “malaria” based on one such a symptom alone.

Some symptoms were specific to particular local *malarias.* For example, breathlessness in Kenya; paleness in central Ghana; swollen legs (among the Nankam) and abdominal pains (among the Kassem) in northern Ghana. Across the sites, no specific symptoms were described for pregnant women compared to other segments of the population. However, vomiting in northern and central Ghana and Kenya, and weakness in both Ghana sites played more prominent roles during pregnancy. Given the wide variety of symptoms associated with *malaria*, respondents in Ghana and Malawi explained that the experiences depended on each individual:

I (interviewer): The poa that you talked of: if someone has *malaria,* how does she/he know that she/he has poa?

R3 (respondent 3): When you feel dizzy and you also vomit, it means that you have *malaria*. At times, you may vomit yellow, which tells you that you have poa. (…)

R2: As for poa*,* we all experience it in a different way because we don’t all have the same system. There are some who have itches on their bodies when they have poa. In addition to that, some don’t vomit, they rather defecate yellowish but watery faeces.

I: What do you have to say about that madam?

R4: Yes, what I want to say is that poa comes in different forms. There are some people that, when they begin to have it, they can’t vomit but feel very cold and have stomach pains.

(Northern Ghana. Focus group discussion with female community members)

**Figure 1:**
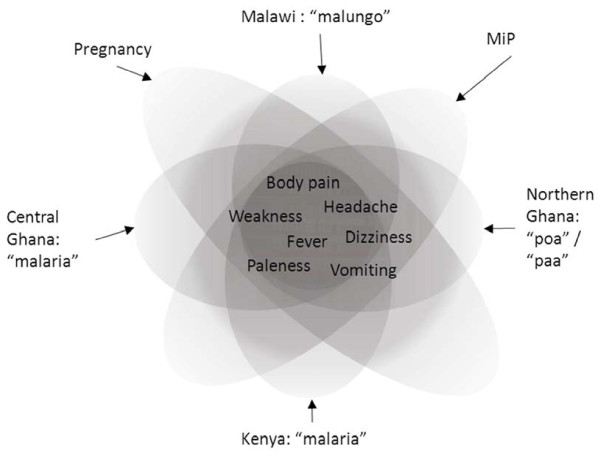
**The overlapping symptoms of local *malarias*, biomedically defined malaria and pregnancy.**

Figure 1 provides a visual representation of how the various local illnesses overlapped with malaria (and pregnancy) in terms of the symptoms experienced. In an abstract manner, the area of each shape represents the range of symptoms that were attributed to each illness and the symptoms most commonly mentioned across the sites are listed in the central overlapping area. In light of the varied descriptions of each illness across the different sites, and the reports of the individual nature of symptoms, this is inevitably a simplification. To encapsulate some of the variation and uncertainty that surrounded the illnesses, each shape is opaque.

At every site, mosquitoes were identified as the main cause of each locally defined illness that overlapped with malaria. Respondents also frequently mentioned poor hygiene and, in some cases, there was a clear link between a lack of cleanliness and mosquitoes, such as stagnant water, bushes around the compound, mosquitoes on the walls, but, in others, there was no such link: dirty cooking utensils, uncovered food and houseflies, cold food and/or, untreated drinking water. At all the sites, a minority of women however reported not knowing the cause of the various illnesses that overlapped with malaria. Exposure to cold and rain was occasionally considered to influence one’s chances of getting “malaria” in Kenya and “malaria” or “fever” in central Ghana, and Malawian respondents also associated hard work with “malungo”. Some food types were also mentioned as causes of the “poa” or “paa” in Ghana: some vegetables and, especially, sweet foods in the north and oily and spicy food at the central site*.*

**Figure 2:**
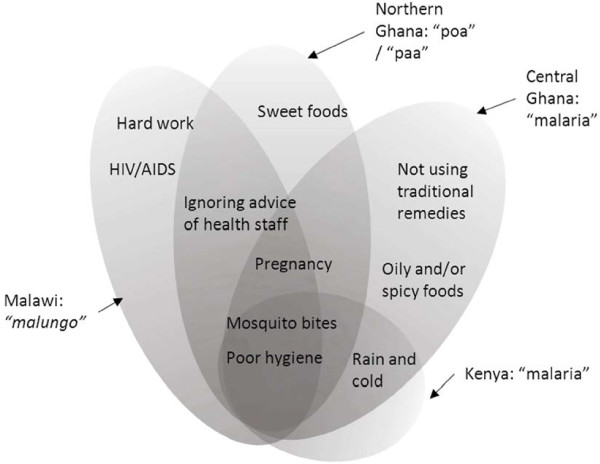
**The overlapping causes of local *malarias.***

At three of the four sites (not Kenya), pregnancy was also generally seen as a direct contributory factor to bouts of *malaria* (see Figure 2). For some informants, pregnant women experience these illnesses (and sickness in general) if they neglect the pregnancy: missing ANC appointments and ignoring health workers’ advice in Ghana and Malawi; or not using pregnancy-specific traditional medicine in central Ghana.

Pregnancy brings about a lot of things. It can give you headache and you sneeze. You feel different in your body. You might vomit. The vomiting is there, but every [type of] vomit means something and yellowish vomit means malaria. Maybe you feel a sour taste in your mouth: this is malaria. I did not have this before I was pregnant, but now I have all these symptoms. I sneeze, and cough, and vomit yellowish, and after eating I throw-up the food.

(Central Ghana, pregnant woman 37 years old, three children)

In Malawi, HIV was also viewed as a provoking the illness that overlapped with biomedically defined malaria, “malungo”.

I: Apart from mosquitoes and general body pains, are there other things that cause malungo?

R: What causes malungo is if you have HIV. You just see malungo frequently, so you wonder and you go to the hospital for a test to see how you are, whether you are ok. Then they find the virus, so you are aware that the malungo is a result of the HIV.

(Malawi. Group discussion with female community members)

Apart from in extreme circumstances, the local *malarias* and bouts of these illnesses during pregnancy were not associated with witchcraft at any of the sites.

### Vulnerability to *malaria* in the context of pregnancy risks

At all the sites, pregnant women, along with children, were generally viewed as particularly vulnerable to *malaria.* Individually, however, women were more uncertain about their relative risk whether pregnant or not, and not all viewed themselves as more likely to suffer *malaria* when pregnant. Among the women with previous experience of *malaria* when pregnant or not, the bouts during pregnancy were generally considered to be more severe, but there were exceptions, for whom experiences of severe *malaria* outside of pregnancy had led them to consider it worse than their experiences when pregnant.

The results of the free listing and sorting exercises (Table 2) showed that the illnesses that overlapped with biomedically defined malaria were amongst the most cited problems: the most cited in Kenya, Malawi and northern Ghana, and third most cited in central Ghana. There was a clear consensus across the sites that *malaria* was the most dangerous problem for pregnant women. *Malaria* was also considered frequent during pregnancy at all the sites except central Ghana. The lesser relevance of the locally defined illnesses in the narratives of pregnant women in central Ghana was indeed later confirmed during in-depth interviews: an important group of women, especially the youngest pregnant women, knew nothing about the disease and the majority reported never having experienced, whether pregnant, or not.

**Table 2:** Health problems that pregnant women suffer (free listing and sorting)

	**Central Ghana**	**Northern Ghana**	**Malawi**	**Kenya**
**Most cited**	Vomiting	“Poa” / “Paa”	“Malungo”	“Malaria”
	Weakness	Vomiting	Vomiting	Backache
	“Malaria”	Weakness		Headache
**Considered dangerous**	“Malaria”	“Poa” / “Paa”	“Malungo”	“Malaria”
	Bleeding	Abdominal pain	Anaemia	“Lack of blood”
	Weakness	“Lack of blood”	Vomiting	Abdominal pain

Vomiting, weakness and headache were often cited as problems for pregnant women across the sites, but were also associated with the various local *malarias*. Indeed, there was a reported overlap between the symptoms of pregnancy and those of mild bouts of *malaria*: headache, weakness, paleness, vomits, lack of appetite, fever and even “malaria” were considered possible symptoms of pregnancy. To differentiate between the symptoms as signs of pregnancy or bouts of *malaria*, respondents referred to the severity of symptoms.

I: How would a woman know that she’s pregnant?

R: Her skin changes; she gets malaria*;* she sleeps a lot; she becomes lazy.

(Central Ghana. In-depth interview with an opinion leader)

I: So how do you differentiate between the signs of malungo and those that are just the signs associated with being pregnant?

R: We look at the seriousness of the disease; so you can tell that this is malungo because it is serious, or they are just signs associated with pregnancy because they are not so serious.

(Malawi. Group discussion with community women).

Pregnant women’s greater risk of suffering one of the *malarias* was mostly attributed to their general vulnerability to disease: pregnant women are “soft”, “weak”, “prone to a number of things”, “two in one”, “carry something inside that means they always have a high temperature”, etc. Women’s inability to meet their dietary needs and multiple poorly spaced pregnancies were considered to compound the weakness of pregnant bodies. Although pregnant adolescents were not seen as particularly vulnerable to *malaria*, their social vulnerability was stressed during the interviews: dropping out of school, terminating the pregnancy (often clandestinely), being rejected by their families, and lacking economic resources were some of the risks mentioned for unmarried pregnant adolescents. Adolescents also had least knowledge of these and other diseases during pregnancy and – as was reported and observed – depended most on others (husbands/“the man who impregnated them”, mothers and other relatives, husbands’ family) to take care of their pregnancies.

### Effects

In general, pregnant women and community members acknowledged that the *malarias* had generally negative effects on women’s health during pregnant and that of their unborn children. If left untreated, they considered that the symptoms would remain, and that they posed a risk to the life of the pregnant women or the unborn child. Indeed, community members saw the association with death as the main reason for considering *malaria* to be the most serious problem during pregnancy. Similarly, miscarriage, stillbirths and preterm delivery were the most commonly mentioned consequence of the various *malarias* in Ghana and Malawi. In Kenya, this direct link, although less common, was not unheard of.

I: So before when we were talking you said that you had suffered malaria during your pregnancies - can you tell us what the symptoms were?

R: Headache, low blood and then my body was turning yellowish. So, when I came back after one week, I miscarried

I: You think the malaria led to the miscarriage?

R: Yes, I think so.

(Kenya. In-depth interview with a pregnant woman, 24 years old, one child)

**Figure 3:**
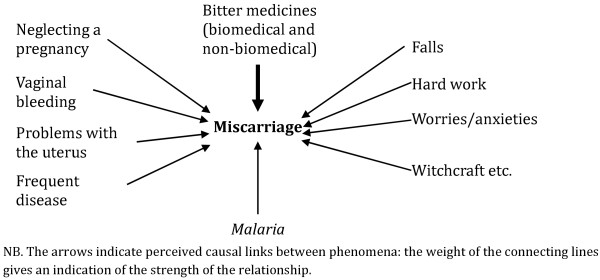
Local explanations of miscarriage (combined across the four sites).

Further probing about the causes of miscarriage illustrated that the local *malarias* were among many other potential causes and was not necessarily the most relevant (Figure 3). At all sites, it was considered that *malaria* and frequent sickness in general played a role, but vaginal bleeding, problems in the uterus, worries, hard work and falls during pregnancy were also important causes of miscarriage. Using bitter traditional or biomedical medicines was acknowledged as the most relevant cause of miscarriage in Ghana and Malawi. Indeed, at these sites, the link between self-medication with either traditional or biomedical drugs and miscarriage was a key message during the health talks given in health facilities when women attended ANC. Moreover, in northern Ghana, there were several reports of pregnant women who had been administered anti-malarials to treat malaria but had miscarried. They had however blamed the drugs and not the illness for the miscarriage. In more general terms, it was also reported that poor ANC attendance or ignoring the advice of ANC staff could lead to miscarriage. Witchcraft was often suspected to have caused a miscarriage, especially in the case of multiple miscarriages. In central Ghana, *asram,* a specific supernatural illness that attacked the babies though their mothers, was also cited as a cause of miscarriage.

Previous severe bouts of *malaria* suffered during pregnancy or by their infants prompted women to link them with anaemia/“lack of blood”. Across all the sites, local terms were directly translated as “lack of blood”, the condition which closely approximated the biomedically defined anaemia, in terms of symptoms, particularly with regard to paleness and lethargy. However, because there were some differences (as is detailed below), the italicized *lack of blood*, is used to differentiate local from biomedical understandings. For many informants, the link was so obvious that they would not mention it until prompted by the researchers, especially in central Ghana, where anaemia-associated paleness was considered one of the main symptoms of *malaria* amongst children and pregnant women. The causal link was however bidirectional: informants at all sites, particularly in Ghana and Malawi, regarded the local *malaria* as a cause of *lack of blood* largely because “fever dried blood” and to a lesser extent because “mosquitoes sucked” blood; others reported that bouts of *malaria* particularly affect women because they are weak as they have insufficient blood (though this was a vague link in Kenya).

**Figure 4:**
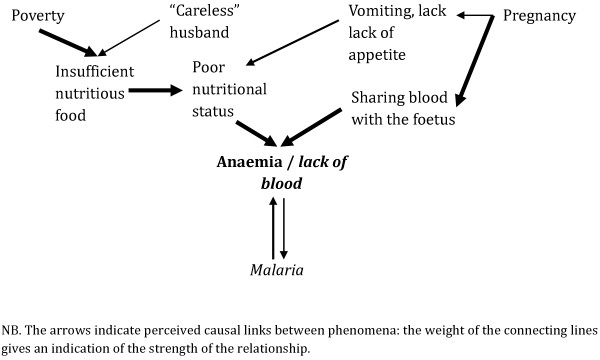
Local explanations of anaemia (combined across the four sites).

The relationship between pregnancy and *lack of blood* was not restricted to the local *malarias* (Figure 4). Blood was rather considered to be a crucial element in pregnancy and foetal development: *lack of blood* was strongly related with the assertion that “pregnant women share the blood with the unborn child”. It was also associated with hazards during delivery, because this is a moment when women “lose more blood”. Women’s diet also had implications for anaemia because food was seen as a key way of avoiding *lack of blood*. Women were said to need better nutrition during pregnancy, but respondents often described how poverty meant that they were often unable to meet this need. Furthermore, pregnancy-related symptoms, such as vomiting and lack of appetite which prevented women from eating, also contributed to this *lack of blood*. Moreover, during interactions with pregnant women as part of ANC, health workers emphasized the relevance of diet during pregnancy. In Malawi, the relationship between *malaria*, pregnancy and anaemia was further complicated by the connections that respondents made between *lack of blood* and HIV/AIDS.

I: What can account for a pregnant woman’s *lack of blood*?

R: The child takes its blood from you so you need to eat a lot and healthily so that, when the child takes blood from you, you’re able to stand the effect and not suffer *lack of blood*.

(Central Ghana. In-depth interview with a woman over 30 years old)

I: Is *lack of blood* common in pregnant women?

R: Pregnant women… it comes, and they have it. Even when the baby is sick, you hear that he has no blood. So where did the blood go? The problem is the mother. Her blood has a bad disease, which she has given the baby. She has bad blood.

I: Can you think about what causes *lack of blood*?

R: HIV is destroying blood.

(Malawi. In-depth interview with a traditional birth attendant)

As previously mentioned, at all the sites, respondents reported that the unborn child was affected by *malaria*. However, only respondents in northern Ghana and in Malawi frequently stated that the negative effects of local *malarias* on unborn children would later emerge as congenital malaria.

I: Let’s suppose you have *malaria* and you did not take any medication, how could you be affected?

R: I might not get well.

I: What about the problems for the child you are expecting?

R: It could be born with malungo*.*

I: So, if born with malungo*,* what are the problems that the child might experience?

R: I might struggle to go to the hospital frequently with him or her.

(Malawi. In-depth interview with a pregnant woman, 17 years old, second pregnancy, childless)

Only two informants linked *malaria* and LBW: one midwife in northern Ghana, and one pregnant woman who was participating in a clinical trial. The weight of the baby was mainly related with nutrition during pregnancy, and thus lack of food, vomiting or lack of appetite and anaemia were considered relevant causal factors. Birth weight preferences were also discussed: in central Ghana and Malawi women preferred newborns to be big as this was considered a sign of health. Whereas, in northern Ghana, women tended to prefer smaller children, which would grow bigger once born, to have an easy delivery. In Kenya, although respondents recognized that babies with a LBW tended to suffer more problems, no particular size preference was stated.

### Risk during pregnancy

The data illustrated multiple relationships between the local *malarias*, other health problems during pregnancy, such as anaemia and HIV/AIDS, and pregnancy itself. However, for the respondents, risk during pregnancy was not only restricted to bodily complaints: a range of social and financial problems emerged during the free listing and sorting exercises and interviews. Such problems cannot be isolated from biomedically (or locally) defined diseases because they have implications for the health of pregnant women. In the communities, poverty was considered to provoke *lack of blood* during pregnancy. As observed and reported by respondents, social relations with husbands and other family members were central to women’s access to resources, and a source of stress, concern and worries that women viewed as possibly harming a pregnancy. Antagonistic relations with relatives or neighbours could also bring about abortions and problems during delivery through witchcraft.

The health implications of social problems were particularly pronounced amongst adolescents: for women of this age group, the consequences of social stigma linked to pregnancy were considered far more relevant than the risk of malaria. Furthermore, on some occasions, this stigma was associated with unsafe abortions and poor access to antenatal care.

I: Which types of problems do adolescents have when pregnant?

R: They will see some problems. For example, they cannot continue with school, they had sex with different people so they don’t know the real father of the baby or they will see that their future has been messed up.

I: Which challenges do they face?

R: They can abort a pregnancy because they suffer a lot of stress (…)

I: How do people in the village talk about pregnancy in adolescents?

R: People always disrespect them or they are less valued in the community.

(Kenya, pregnant woman case study, 24 years old, one child)

Health issues can also affect social relations. For a pregnant woman, an HIV diagnosis could have a critical impact on her marriage, as could recurrent pregnancy loss and infertility. The symptoms of pregnancy (*malaria* or *lack of blood*), such as weakness, (referred by many as “laziness”) could make it difficult for women to work in the fields and this had both financial and relational consequences.

The people with whom you live might think that you deliberately don’t want to work, but you know that it hurts. So he can say that he would not do what he usually does for you because you are being deliberately lazy. You know that the work is yours to do but you lie down and…you cannot do it….They would say that you should get up and work so that your body is smart but you are lying down… You cannot get up. They say that you are doing it deliberately, that you have let the pregnancy overcome you.

(Northern Ghana. Pregnant woman, 27 years old, two children)

This problem appeared frequently during discussions, especially in northern Ghana, but the case studies showed that although women reduced their heavy household duties during pregnancy (such as collecting water and lifting other heavy weights), many continued with chores almost until delivery and then begun again weeks or days afterwards.

## Discussion

Across the four sites, the various local *malarias* overlapped considerably with the biomedical concept of malaria. Nonetheless, with regard to symptoms, vulnerable groups, outcomes and aetiology, this overlap, in the context of pregnancy, was both site-specific and incomplete. This partial overlap meant that respondents occasionally identified an illness as *malaria*, but health professionals did not diagnose malaria. In other instances, health professionals might diagnose malaria, whereas the sufferer did not consider it to be *malaria*. The boundaries between local and biomedical concepts overlap and they are not clearly defined (this is represented in an abstract, visual manner in Figure 1), with symptoms varying between respondents and from one episode of illness to the next. In light of the uncertainty surrounding the symptoms of biomedically defined malaria during pregnancy [8,9], in Figure 1 the area that represents biomedically defined malaria is also poorly defined.

These findings resonate with previous qualitative research that has identified a variety of local *malarias* that overlap, often partially, with biomedically defined malaria and malaria during pregnancy [6,26,32-35]. In southern Malawi, previous studies employing qualitative methods at different research sites [35,36] have also described illness(es) that were referred to using the Chichewa term, “malungo”. Moreover, these illnesses share some (but not all) characteristics with the “malungo” that respondents described during fieldwork in Malawi for this research: both were caused by hard work or mosquitoes and the severity varied, but, for example, in the previous studies, respondents viewed “malungo” during pregnancy as less dangerous. Within Malawi, the significance of the disease concept attached to this particular Chichewa term has therefore seemingly changed over time or it differs at these research settings (separated by hundreds of kilometres). This heterogeneity underlines how, when carrying out research (or implementing interventions) in the same or nearby sites over time or in other settings where the same terms are used, the meaning of such terms – in terms of the attached illness concepts – cannot be taken for granted; nor can such terms be unproblematically translated as malaria.

Respondents across all the sites considered the local illnesses that overlapped with biomedically defined malaria, to be the most dangerous disease during pregnancy and pregnant women were regarded as one of the most vulnerable groups. These findings coincide with previous studies in Zambia [37], Malawi [38], Tanzania [39], Ethiopia [40], Kenya [41], Uganda [34,35,42,43], Nigeria [44], Senegal [45] and the Gambia [46]. At all sites except central Ghana, across the different categories of respondent, the local *malaria* was viewed as common during pregnancy.

The relationship between local illness and biomedically defined malaria was however further complicated by women’s experience of pregnancy. As other authors have highlighted [26,32,33,35,42,45,46], many symptoms linked to *malaria*, including headache and fever, were considered to be *normal* symptoms or problems related to pregnancy. In some instances, pregnancy was also offered as an explanation for bouts of *malaria*. Furthermore, whether women labelled such episodes as about of Malaria depended upon the seriousness of symptoms (and their impact on their daily lives). Hence local *malarias* and pregnancy are interwoven domains of experience.

Local explanations of pregnancy loss, anaemia and LBW were complex and, in that sense, they resonate with the findings of biomedical research [14-20]. However, among their multiple causes, *malarias* were never considered the most important factor. Emphasis was also often placed on their social underpinnings, such as poverty, hard work during pregnancy, an unhelpful husband, or psychological strain and general ill health. Moreover, as previous anthropological research has emphasized [47,48], the findings demonstrate the interrelation between social, economic and health problems during pregnancy.

### Framework for MiP interventions

Given the complex relationships between experiences of pregnancy on the one hand and malaria/*malaria* on the other, ANC must address the web of pregnancy-related problems that women often perceive as interrelated. It is therefore essential that WHO-recommended procedures for ANC [49] promoting an integral care strategy are followed and that the fragmentation of ANC into in multiple isolated procedures (as is sometimes perceived in practice) is avoided.

Comprehensive ANC is essential to improve safe motherhood and ensure effective malaria prevention and control, but other social and economic strategies are also needed to fully address pregnant women’s vulnerabilities. This is especially relevant in the case of adolescents, for whom the social risks of pregnancy eclipse their higher vulnerability to, and greater morbidity and mortality as a result of, MiP. The social consequences and the stigma of adolescent pregnancy must also be addressed to ensure ANC attendance and access to MiP interventions [7,35,50].

### Health messages about malaria and MiP

The results show that some messages about malaria and pregnancy are nowadays part of local ideas about health and disease: mosquitoes are recognized as the main cause of *malaria* at all the sites; lack of hygiene is considered an important cause of disease, even though it is sometimes viewed as a cause of *malaria*[23]; women are aware that they should consume nutritious food to prevent anaemia during pregnancy; and self-medication is often viewed as a risky practice that can provoke miscarriage. Nonetheless, the relevant health messages, and the form in which they are communicated, could be modified to promote greater awareness of MiP and its consequences. Two specific areas seem particularly relevant:

1. Awareness of MiP’s deleterious effects was not universal: miscarriage and congenital malaria were the best known; anaemia was acknowledged by some; but LBW was not mentioned as a direct effect. Consequently, it is important to move from a generic model of the severity of MiP to more pragmatic and specific messages about malaria’s serious implications during pregnancy, focusing on its role in compounding or provoking anaemia, LBW and pregnancy loss.

2. At all the sites, adolescents’ greater risk of MiP was relatively unknown. A broader approach is however necessary to ensure the effective communication of messages about adolescent’s vulnerability to MiP. To reach adolescent before they become pregnant requires incorporating such messages into health education at school. However, given their dependence on other people to access ANC, targeting health messages at adolescents’ is not sufficient: entire communities must rather be aware of these messages and take responsibility for the success of health promotion.

### Site specificities and their implications

Other interventions and health messages require tailoring to local settings. In central Ghana, it is important that women learn to identify MiP and raise their awareness that it is a common problem during pregnancy. On the other hand, the prominence of weakness and paleness as *malaria* symptoms reveals a close link between anaemia and *malaria* that can facilitate specific messages regarding the repercussions of MiP (as described in other studies conducted in a nearby area [49]).

In northern Ghana, health providers should be aware of the local links between *malaria* and sweet-tasting foods, and local preferences for delivering LBW babies in order to avoid misunderstandings and negative reactions to their advice. The association of *malaria* with consuming sweet-tasting foods and drinks during pregnancy is a source of misunderstanding. Some women distrust and therefore ignore nurses’ advice about eating something sweet in order to avoid the side effects of malaria treatment.

In an earlier article on the possible contributions from the social sciences to research on malaria in pregnancy, the authors cite an example from Burkina Faso of women who prefer “the baby to grow after giving birth instead of before” and thus having bigger babies could be considered an undesired effect of malaria treatment [51]. During data collection, a similar preference for delivering small babies was only identified in the neighbouring northern Ghana. This preference needs to be addressed because it probably interferes with some of the advice regarding healthy nutrition. Nevertheless, given the lack of association between LBW and local *malaria* concepts*,* it does not seem to affect acceptance of MiP treatment or prevention.

In Malawi, there were associations between perceptions of HIV and *malaria* that need to be taken into account for health campaigns. HIV interacts with and complicates malaria’s epidemiology and pathology [1,52,53] as well as the social context and understanding of disease during pregnancy. The shared understanding of *malaria* and HIV could, in part, explain the common association between MiP and congenital malaria, which, given its rare symptomatic presentation, is notable [12]. It is related to local understanding of illness transmission during pregnancy, which, in Malawi, has probably been reinforced by the widespread health education on the prevention of mother-to-child HIV transmission.

### Strengths and limitations

The use of qualitative methods, in combination with long-term data collection, has enabled the analysis of understandings of MiP and their contextualization within a background of pregnancy-related problems. The comparative approach, using parallel techniques and topics of research, also drew attention to both the similarities and differences between the sites, which could have otherwise been taken for granted.

This article has presented no specific data on health professionals’ views of MiP their perceptions of MiP require further analysis because, as they are instrumental to the supply of effective MiP interventions. Finally, the inclusion of an additional site with low seasonal malaria transmission would have enabled greater assessment of how the epidemiological context influences local perceptions of MiP.

## Conclusion

At each study site, complex relationships between local concepts of *malaria* that overlapped with biomedically defined malaria and the symptoms of pregnancy were identified. Pregnancy was often considered to provoke *malaria*; *malaria* was also seen as a symptom of pregnancy; and many of the symptoms of *malaria* and pregnancy overlap. Treatment and prevention for MiP should therefore be delivered as part of a comprehensive ANC programme and fragmentation of ANC into separate disease-specific interventions avoided. However, it is important to avoid expanding MiP interventions to the detriment of other core health issues, such as anaemia and miscarriage, some of which can be linked to MiP but whose aetiologies are more complex. This study also underlines how when designing MiP prevention and control strategies, in addition to the changing contexts of malaria transmission and drug resistance, it is necessary to take into account the specific and *fluid* local relationships between malaria and illness during pregnancy. These relationships are especially relevant for the design of adequate health education that should form part of the promotion of any new health intervention.

## Competing interests

The authors declare that they have no competing interests.

## Authors’ contributions

AM contributed to the overall study design; supervised and assisted with data collection in Ghana and Malawi; analysed the data from these sites; prepared the first draft of the manuscript and contributed to its revision based on comments from co-authors. CP contributed to the overall study design; supervised and assisted with data collection in Kenya; analysed the data from this site; provided comments on the first draft of the manuscript and contributed to its revision based on the comments from co-authors. LM collected data at the Malawi site; revised the manuscript and provided comments. SC collected and supervised data collection in northern Ghana; revised the manuscript and provided comments. NAA collected data in central Ghana; revised the manuscript and provided comments. FW collected data in Kenya; revised the manuscript and provided comments. AH supervised data collection in northern Ghana; revised the manuscript and provided comments. PO supervised data collection in Kenya; revised the manuscript and provided comments. LK supervised data collection in Malawi; revised the manuscript and provided comments. HT supervised data collection in central Ghana; revised the manuscript and provided comments. RP conceived and designed study; obtained project funding; provided comments and contributed to the revision of the manuscript based on comments from all co-authors. All authors read and approved the final version of the manuscript.
